# A 19th Century Stormwrecked Black‐Capped Petrel From Vermont Offers Insight Into Historical Vagrancy Processes

**DOI:** 10.1002/ece3.70846

**Published:** 2025-02-05

**Authors:** Oliver W. Patrick, Max Chalfin‐Jacobs, Arthur Lyu, Jody Smith, Ellery Foutch, Alexis M. Mychajliw

**Affiliations:** ^1^ Department of Biology Middlebury College Middlebury Vermont USA; ^2^ Program in Environmental Studies Middlebury College Middlebury Vermont USA; ^3^ Sciences Technical Support Services Middlebury College Middlebury Vermont USA; ^4^ Department of American Studies Middlebury College Middlebury Vermont USA

## Abstract

Specimens stored within museum collections are increasingly leveraged to reconstruct historical baselines to both decipher the legacies of past anthropogenic impacts and anticipate the consequences of future climate change on species distributions. However, the research significance of such collections can be severely constrained based on their curation histories, resulting in data being forgotten, if not lost entirely. In this Nature Note, we report the unexpected presence of a mislabeled Black‐capped Petrel ( *Pterodroma hasitata* ) specimen in the historical Middlebury College Vertebrate Natural History collection, potentially representing the rediscovery of a lost specimen reported from Vermont following the 1893 New York City Hurricane. We conducted archival research at multiple institutions to substantiate the reporting of a Black‐capped Petrel specimen that was “missing” from Vermont in 1893, as noted in the Vermont Breeding Bird Atlas. We further substantiated the 19th‐century age of this specimen through X‐ray fluorescence analysis of mercury and arsenic of more than 200 whole bird bodies and feathers across the majority of the Middlebury College collection as part of an environmental health and safety assessment. This record expands the known vagrant range of the Black‐capped Petrel. This research likewise highlights the critical role of small museum collections play in piecing together historical datasets and informing modern conservation, emphasizing the importance of their preservation and digitization.

## Graphical abstract

Small historical museum collections can provide unexpected data points in telling the story of how climate change has impacted species. We report a (re)discovered Black‐capped Petrel ( *Pterodroma hasitata* ) from the 19th‐century Middlebury College Vertebrate Natural History collection and confirmed its late 1800s Vermont origin through a combination of archival and non‐chemical research. This individual likely represents the missing storm wrecked specimen associated with a 1893 vagrant record from Vermont and aligns with the 1893 New York City Hurricane.

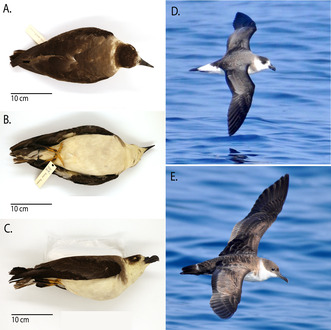

## Abstract (es)

Los especímenes guardados en colecciones históricas de museos cada vez más se apalancan para revertir las líneas de base cambiadas en el contexto de anticipar el cambio climático global e interpretar los legados de impactos antropogénicos pasados. Además, la importancia investigativa de estas colecciones pueden ser limitadas debido a sus historias de curación, que resulte en data olvidada, si no completamente perdida. En este Nature Note, reportamos la presencia inesperada de un espécimen de un diablotín ( *Pterodroma hasitata* ) mal etiquetado en la colección de la historia natural de vertebrados de Middlebury College, un espécimen que potencialmente representa el redescubrimiento de un espécimen perdida reportada en Vermont tras el huracán de la ciudad de Nuevo York de 1893. Realizamos una investigación de archivo en un número de instituciones para corroborar el reportaje del diablotin ‘pérdida’ de Vermont en 1893, como notado por el Vermont Breeding Bird Atlas. Probamos que el espécimen se originó en el siglo XVIII con un análisis de fluorescencia de rayos X de mercurio y arsénico de más de 200 cuerpos completos y plumas de pájaros tras la mayoría de la colección de Middlebury College. Este registro amplía el rango de la vagancia conocida de la especie y aumenta el conjunto de datos para interpretar patrones históricos, modernos, y futuros de *stormwrecking* en esta especie en peligro de extinción. Esta investigación además destaca el rol crítico de colecciones de museos pequeñas en reconstruir data histórica e informar la conservación moderna, enfatizando la importancia de su preservación y digitalización.

## 1 Introduction

**FIGURE 1:**
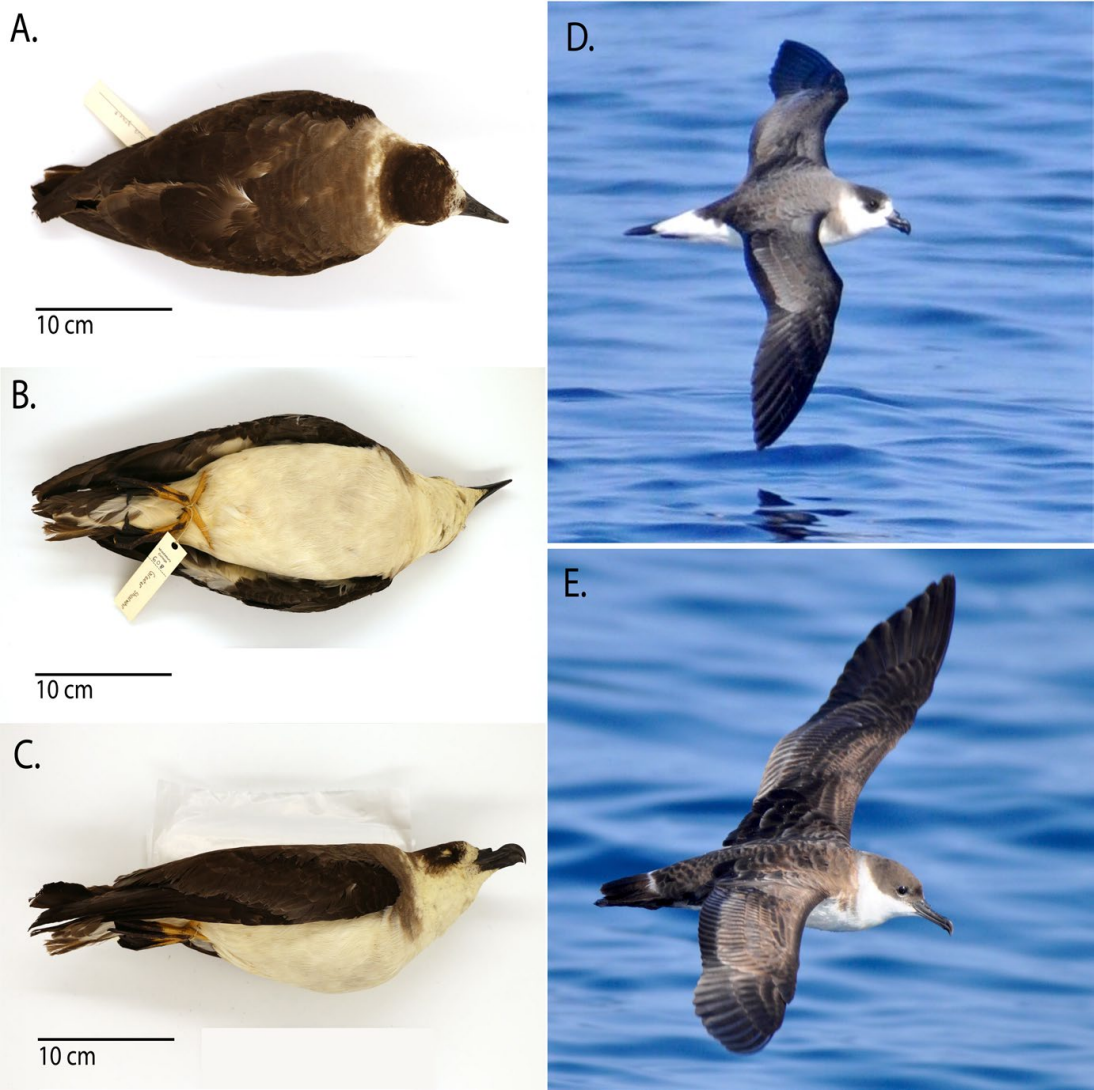
(A) Dorsal, (B) ventral, and (C) right lateral view of Middlebury College Black‐capped Petrel specimen (#608). (D) Live Black‐capped Petrel photographed off Ocean City, Maryland. (E) Live Great Shearwater photographed off Ocean City, Maryland (all photographs by O.W. Patrick).

**FIGURE 2:**
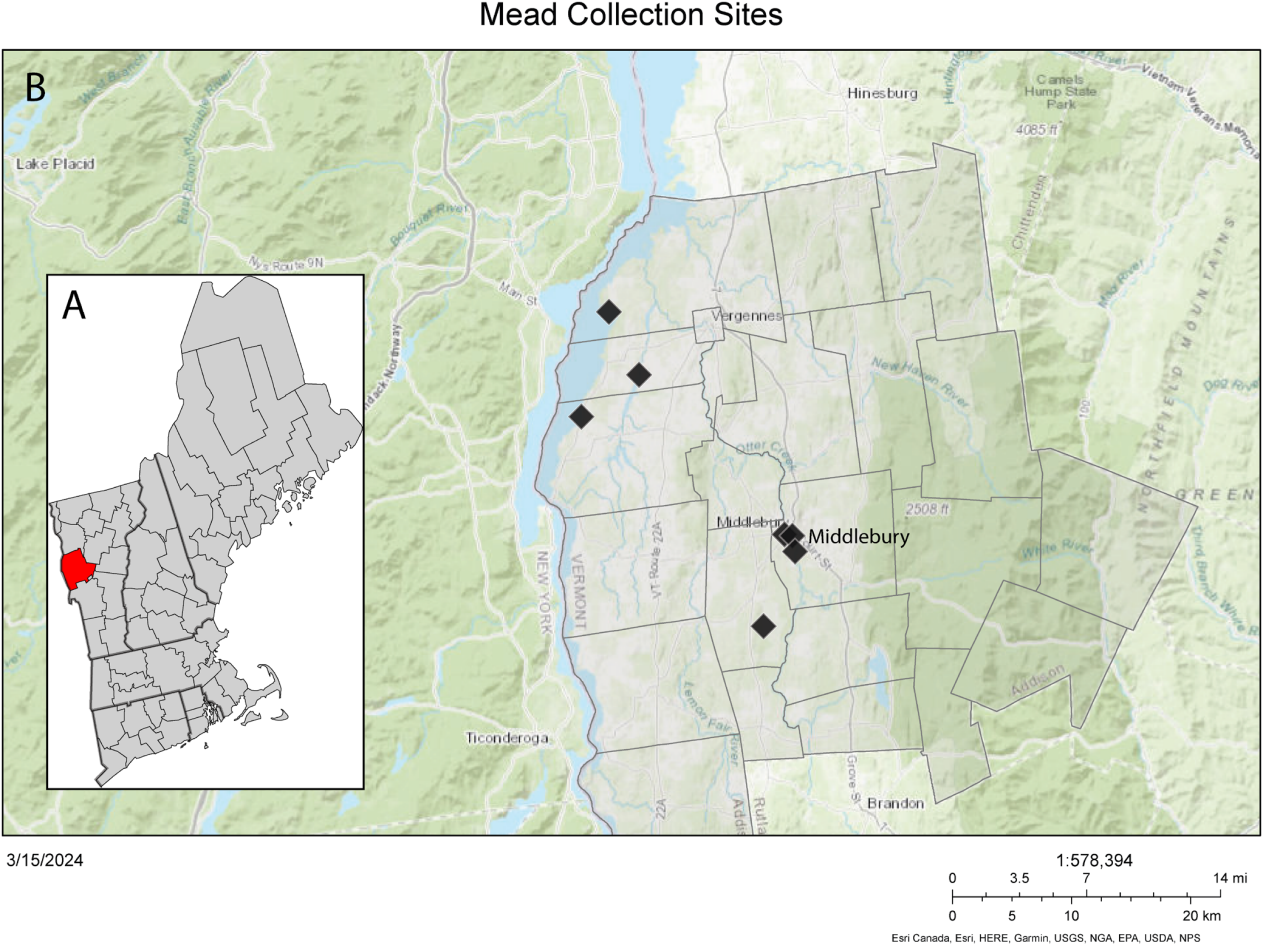
(A) Map of Addison County (red) within Vermont and the larger New England area (adapted from Wikimedia Commons). (B) Key sampling areas represented by the Middlebury College Vertebrate Natural History Collection's Parkhill‐Mead bird specimen collection within Addison County, formed in the late 1800s. Coordinates were plotted from site descriptions based on descriptions within the letters of Albert D. Mead (Mead 1944) and georeferenced using GEOlocate (https://www.geo‐locate.org/).

Global climate change has caused the geographic distributions of numerous bird species to shift, exacerbating conservation challenges already faced by species whose ranges span international boundaries and oceans (Sanderson et al. 2016; Bateman et al. 2020). Disentangling the environmental and anthropogenic factors that determine the extent of species ranges in the present is essential for predicting how those ranges will shift in the future, and researchers are increasingly turning to natural history collections and other sources of historical ecological data to address shifted baselines in conservation (e.g., Tingley et al. 2009; Iknayan and Beissinger 2018; McClenachan et al. 2024). While many of these studies focus on large, digitized collections of global extent (Page et al. 2015), small museum collections house specimens crucial to local‐scale endeavors and can also reveal data tied to local human histories that match local conservation priorities (e.g, Casas‐Marce et al. 2012; Flood et al. 2020; LeFebvre et al. 2023; Olsen et al. 2020). Here, we report the serendipitous (re)discovery of an endangered Caribbean seabird, the Black‐capped Petrel ( *Pterodroma hasitata* ), from the recently restored Middlebury College Vertebrate Natural History collection (Figure 1). The Middlebury College collection is a small 19th‐century museum collection derived from a restricted sampling area of Addison County, Vermont, inland New England, United States (Figure 2). It was formed primarily through the efforts of two local students, Chester Parkhill and Albert D. Mead in the late 1800s. This new locality represents an apparent first occurrence of *P. hasitata* for the state of Vermont and—when placed into appropriate historical ecological context—provides greater insight into how the species may respond to future climate change through increased storm frequency, a known driver of bird mortality (Frey et al. 2009; Hass, Hyman, and Semmens 2012; Wheeler et al. 2021).

The Black‐capped Petrel (known as the Diablotín in the Caribbean) is an endangered gadfly petrel (genus *Pterodroma*) with an estimated population of 1000–2000 mature individuals worldwide (BirdLife International 2018). The IUCN Red List categorizes the species as endangered (B2ab (ii, iii, v) criteria) due to its fragmented range, historical extirpations starting in the 19th century, and ongoing decline linked to habitat loss, invasive species, and hunting (BirdLife International 2018; Satgé et al. 2024). Today, the species is known to breed primarily in high elevation areas on the Caribbean island of Hispaniola, with a small additional breeding population present on Dominica. Breeding in eastern Cuba is suspected but unconfirmed (Leopold et al. 2019; Satgé et al. 2024), and the species is likewise probably extirpated from historical sites on Martinique and Guadeloupe (Satgé et al. 2024). During the nonbreeding season, the species ranges widely across and beyond the Caribbean, and a key foraging area is the western wall of the Gulf Stream along the Atlantic seaboard of the United States (BirdLife International 2018; Satgé et al. 2023). While typically strictly pelagic, a large number of Black‐capped Petrel mortalities have been recorded inland in eastern North America following powerful (Saffir‐Simpson Category 3 or above) hurricanes for over a century—representing stochastic events that could significantly impact an endangered species with such a small population size (Hass, Hyman, and Semmens 2012). Populations remain in long‐term decline, with quasi‐extinction modeled as likely within 100 years (Wheeler et al. 2021); therefore, any source of mortality is a cause for concern.

During a preliminary resurvey of the Middlebury College Vertebrate Natural History collection, which had been obscured in storage for decades and had not been systematically curated, we identified a Black‐capped Petrel specimen mislabeled as a Great Shearwater, *Ardenna gravis* (verbatim labeled as “Greater Shearwater, *Puffinus gravis* ”) (Figure 1). The specimen's tag—clearly a recent retagging by a student worker rather than the original 19th‐century tag—did not describe a date or location and utilized an incorrect English name and a genus predating a 2016 taxonomic revision (Chesser et al. 2016). While many specimens from the 19th century within this collection remain attached to their original labels (with date, locality, and other metadata meticulously noted by the original collectors and preparators), many others were relabeled or relocated by various workers and students over the years of the collection's storage, unfortunately resulting in data loss. As such, we employed several methods to confirm the *P. hasitata* specimen as a 19th‐century Vermont specimen, including (1) archival research using historical sources at Middlebury College, the Smithsonian, Brown University, and the American Museum of Natural History, and (2) non‐invasive chemical analysis to verify the presence of arsenic inherent to pre‐1950s specimen preparation methods. We further contextualize this specimen within the species' past and present geographic distributions as they relate to storm‐driven vagrancy.

## 2 Methods and Results

### 2.1 The 19th‐Century Collection

Middlebury College houses a collection of nearly 500 19th‐century bird specimens (study skins and taxidermied mounts) which have been used occasionally in teaching or otherwise left in storage. A trove of letters, articles, and college proceedings pertaining to the collection has been maintained by Middlebury College Archives and Special Collections. Middlebury student Frank Hall Knowlton donated the first few specimens from 1880 to 1884 (Knowlton 1882; The Museum 1898) and subsequently trained two local high school students, Chester H. Parkhill (1867–1890) and Albert D. Mead (1869–1946), in specimen preparation (Mead 1944). The vast majority of the Middlebury specimens were collected between 1884 and 1890 by Parkhill and Mead from within the small geographic radius of Addison County, Vermont (e.g., Parkhill 1888; Memorial Collection of Birds 1939) (Figure 2). Active specimen acquisition effectively ceased after Parkhill's death in 1890 (Middlebury Register 1890), and these specimens were donated to the college. Seven well‐labeled specimens with a clear provenience of Ithaca, New York, were added to the collection in 1957 by David and Eunice Van Vleck. A further few specimens represent birds recently acquired from 1983 onward under the supervision of former professor Steve Trombulak (S. Trombulak, personal communication, January 16, 2024) and are visibly different in preparation and preservation state.

### 2.2 Species Identification

We identified the specimen as a Black‐capped Petrel based on the presence of several diagnostic external features. A white uppertail and a comparatively short, deep bill comprehensively eliminated the erroneous labeling of the specimen as a Great Shearwater (*Ardenna gravis*) and instead diagnosed the specimen as a gadfly petrel (*Pterodroma*). Of gadfly petrels known from the western Atlantic, Trindade Petrel ( *P. arminjoniana* ), Cape Verde Petrel ( *P. feae* ), Desertas Petrel ( *P. deserta* ), and Zino's Petrel ( *P. madeira* ) were eliminated due to the specimen's dark cap and white uppertail. Finally, the Bermuda Petrel ( *P. cahow* ) was eliminated due to a combination of characteristics, including the level of contrast between the dark cap and paler mantle, the extent of the white uppertail, and presence of a pale collar.

The specimen's plumage is consistent with the “Dark Form” (Dark‐faced Form) of Black‐capped Petrel, a form that, alongside the “light form” (white‐faced form) of the species, has been suggested as potentially deserving subspecific or even specific status in light of differences in genetic structure, plumage, measurements, range, and molt timing (Howell and Zufelt 2019). Dark form individuals have a more southerly nonbreeding range than light form birds, favoring the western edge of the Gulf Stream within the Carolinian marine ecoregion, whereas light form individuals utilize cooler and deeper waters to the north, within the Virginian marine ecoregion (Satgé et al. 2023). Photographs of the specimen were sent to K. Sutherland, an expert on *P. hasitata* morphology and identification (e.g., Satgé et al. 2023), who described the specimen's plumage as consistent with an adult bird. However, as the specimen's wings do not open, we could not observe molt features that would aid in determining time of year of collection (K. Sutherland, personal communication, March 29, 2024).

### 2.3 Archival Research

We conducted archival and records research to obtain the surviving correspondence of Albert D. Mead held at Middlebury College (Hitchcock et al. 1950), the Smithsonian Institution Archives Division of Birds Records, and the Albert D. Mead Papers at Brown University (Mead 1890‐1937a, Mead 1980‐1937b, Mead 1980‐1937c). In a 1939 letter, Mead noted that “[b]oth the specimens and the notes concern almost exclusively the area of Addison County, and the four academic years 1886–1887 to 1889–1890” (Mead 1944). Citing his own 1890 thesis (currently unlocated), he described the area of collection as following: “The center of operations may be considered as the Middlebury College Campus. Occasional expeditions have been made in all directions in search of birds and facts. The favorite localities are the campus, the ‘Railroad Swamp,’ a small low wood just opposite the ‘mile bridge’ on Otter Creek (one mile South of center of Village), the ‘Big Swamp’ lying East of Cornwall, Dead Creek already located, and Lake Champlain in Addison and in the vicinity of Button Bay in Panton” (Mead 1944). No mentions of a Black‐capped Petrel, Great Shearwater, storm wrecked seabirds, or hurricanes were discovered in these surviving correspondences of Albert D. Mead, which, it must be remembered, are themselves an incomplete subset of all correspondences that once took place.

**FIGURE 3:**
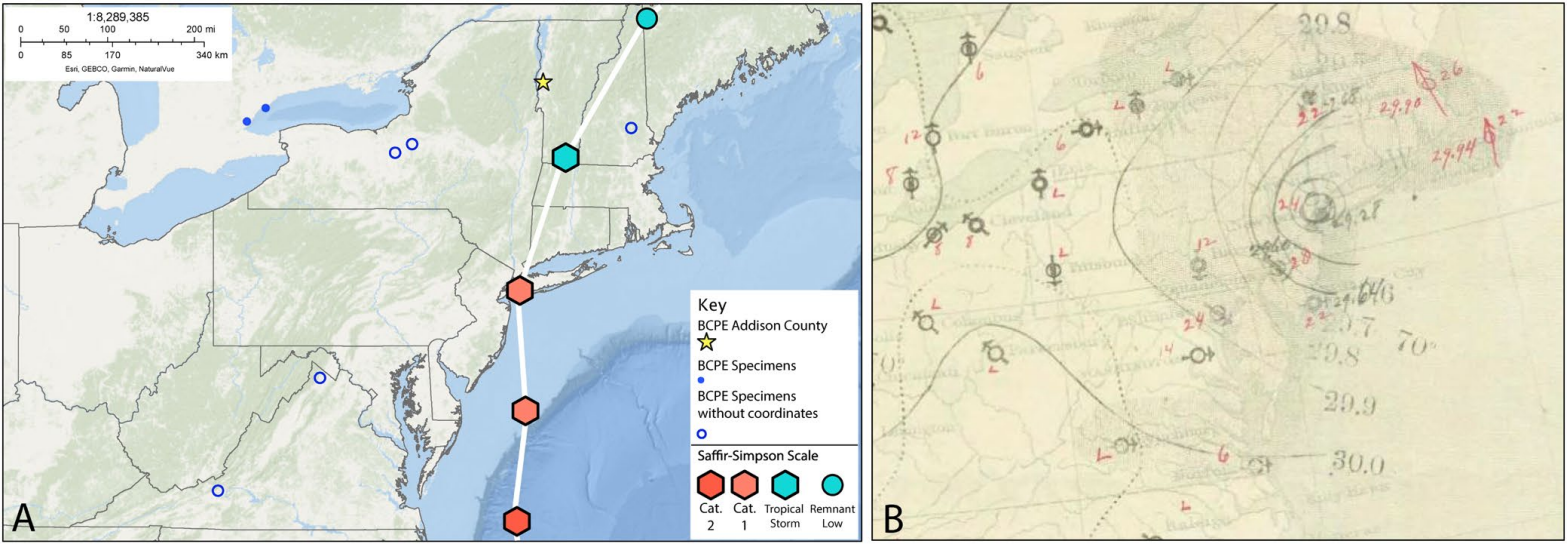
(A) Black‐capped Petrel (BCPE) records associated with the 1893 New York City Hurricane. Hurricane trajectory and records courtesy of National Hurricane Center (2009), path adapted from Wikimedia Commons. (B) Historic map of 1893 New York City Hurricane (NOAA Central Library Data Imaging Project, Public Domain). Maps made in ArcGIS Online (ESRI) with base map “Oceans”.

Through our search of historical documents, we confirmed the presence of a storm wrecked Black‐capped Petrel in Vermont in late August 1893. This information was communicated privately to Joel Asaph Allen of the American Museum of Natural History, though no specific location within Vermont was provided (Allen 1894, 1904). A search of J.A. Allen's correspondence in the Archives & Ornithology Department records at the American Museum of Natural History, including copies of outgoing correspondence in numerous letter books from August 1893 to September 1894, did not yield any further information (Allen 1893). Glover M. Allen later included this record in a treatise on New England fauna (Allen 1909). According to Senior Conservation Biologist Kent McFarland at the Vermont Center for Ecostudies, which maintains the Vermont Atlas of Life and the Vermont Breeding Bird Atlas, this record was not accepted by the Vermont Bird Records Committee as of 1986 due to G. M. Allen's description of the bird as “specimen missing” (K. McFarland, personal communication, April 3, 2024). It is therefore possible that the Middlebury specimen represents this missing specimen. The record was noted in the context of the 1893 New York City Hurricane, a Saffir‐Simpson Category 3 tropical cyclone that struck New York City and moved up the Hudson Valley on August 24 of that year (Coch 2019). In the wake of this event, seven other vagrant Black‐capped Petrels were reported in the region (Allen 1894), including from Oneida Lake, New York on August 28 (Bagg 1894; Allen 1894), Pittsfield, New Hampshire on August 30 (Harvard University M and Morris 2024), Blacksburg, Virginia on August 30 (Smyth 1893), Winchester, Virginia on August 31, Cayuga County, and New York in September (Eaton 1910) and October 30 in Toronto, Ontario, Canada (Allen 1894) (Figure 3).

We did not recover any mention of a Black‐capped Petrel or Great Shearwater in their surviving notes available to us (Parkhill 1888; Perkins and Howe 1901). We therefore cannot discount the possibility that this specimen was an extraneous addition to the collection and provide this note as a conservative hypothesis based on the best available evidence at hand. Seabird storm wrecking is stochastic, and downed seabirds are often recovered by nonornithologists (e.g., Smyth 1893; Foster 1895; Lindahl 1899) before being presented to local specialists for identification and curation. This possibility therefore does not contradict a local origin for the specimen. However, there is no historical record or quantitative evidence from the specimen to suggest this, and further, no record of bird specimen loans from any other institutions ever being held by Middlebury College.

### 2.4 Geographic Range and Historical Vagrancy Records

**FIGURE 4:**
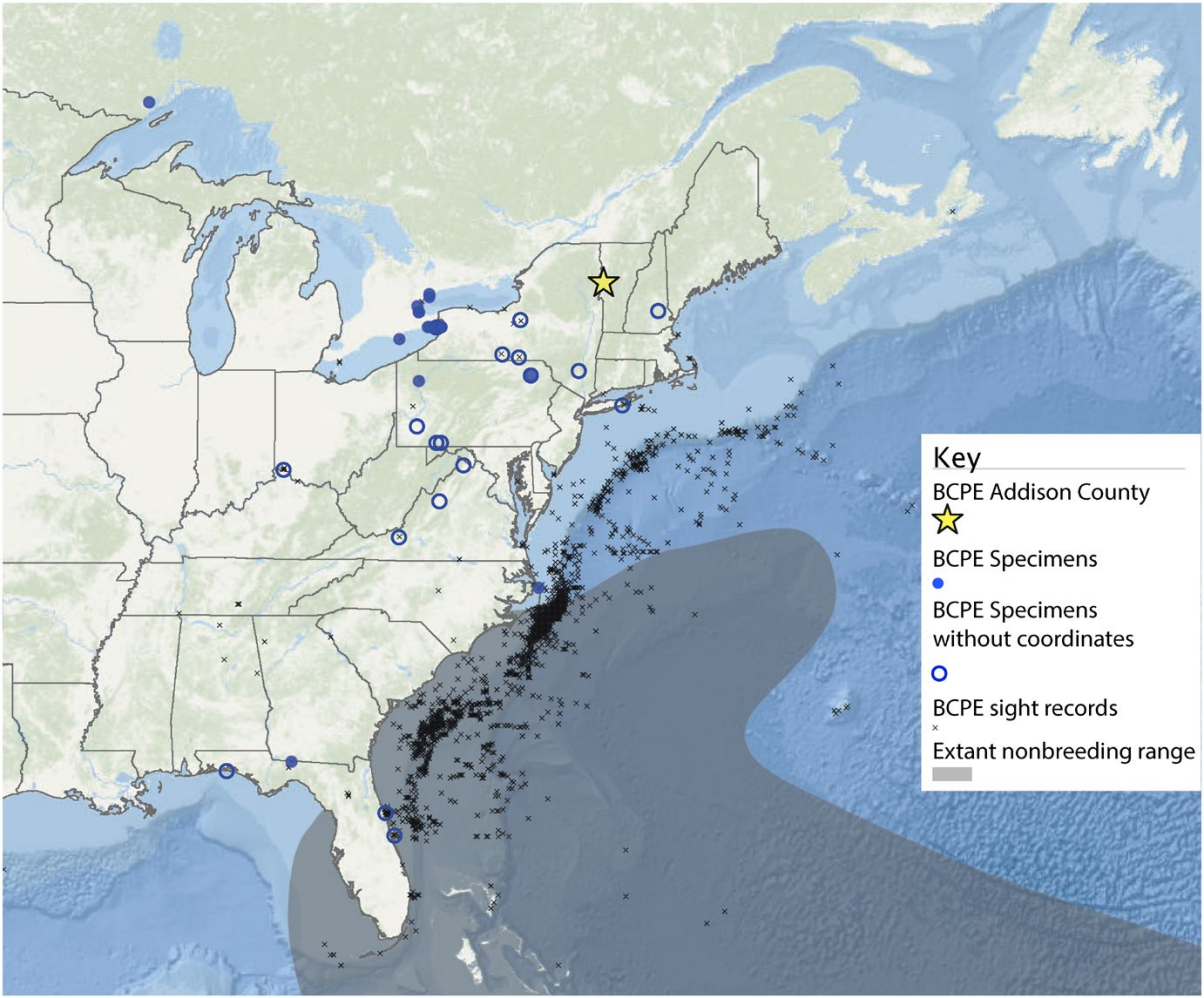
Black‐capped Petrel (BCPE) records from Addison County (star) and other North American localities accessed via GBIF (blue circles) (GBIF.org 2024). BCPE range from the IUCN Red List (BirdLife International 2018). Note that the full range of the species extends southwards into the Caribbean and is not shown here.

We queried the Global Biodiversity Informatics Facility (GBIF) for historical (i.e., cataloged museum specimens) georeferenced occurrences and present‐day observations (i.e., research‐grade iNaturalist photographs and eBird records) of the species (GBIF.org 2024), and mapped these records using ArcGIS Online. Any specimen cataloged on GBIF that possessed location information below a state/provincial level but lacked exact coordinates was manually mapped using GEOlocate following standard procedures (https://www.geo‐locate.org/) (see usage in Hill et al. 2009). Specimen records from offshore locations or outside North America were not included in our map given the geographic focus of our study. A current range map of the Black‐capped Petrel from the IUCN Red List (BirdLife International 2018) was incorporated for comparison (Figure 4).

**FIGURE 5:**
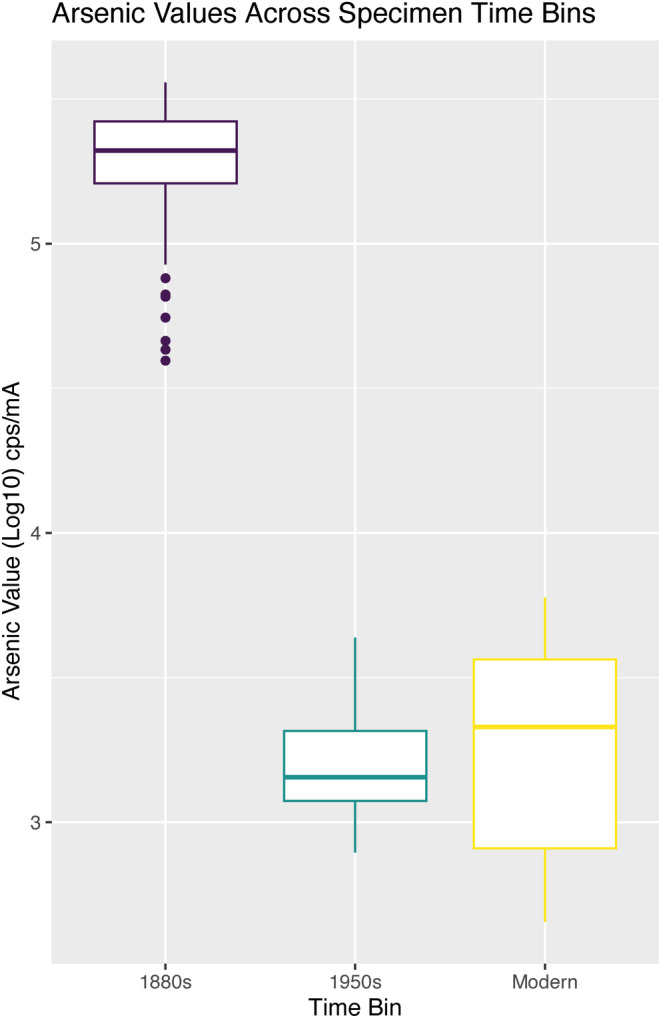
Arsenic levels (log10 cps/mA) across the 1880s, 1950s, and modern (1983–2019) bird collections in the Middlebury College natural history collection (*n* 1880s = 198, *n* 1950s = 7, *n* modern = 7). Values reported are in cps/mA, counts per second per milliamp, which is a measurement of peak intensity. Values are presented as log10 due to the magnitude of differences between readings.

**FIGURE 6:**
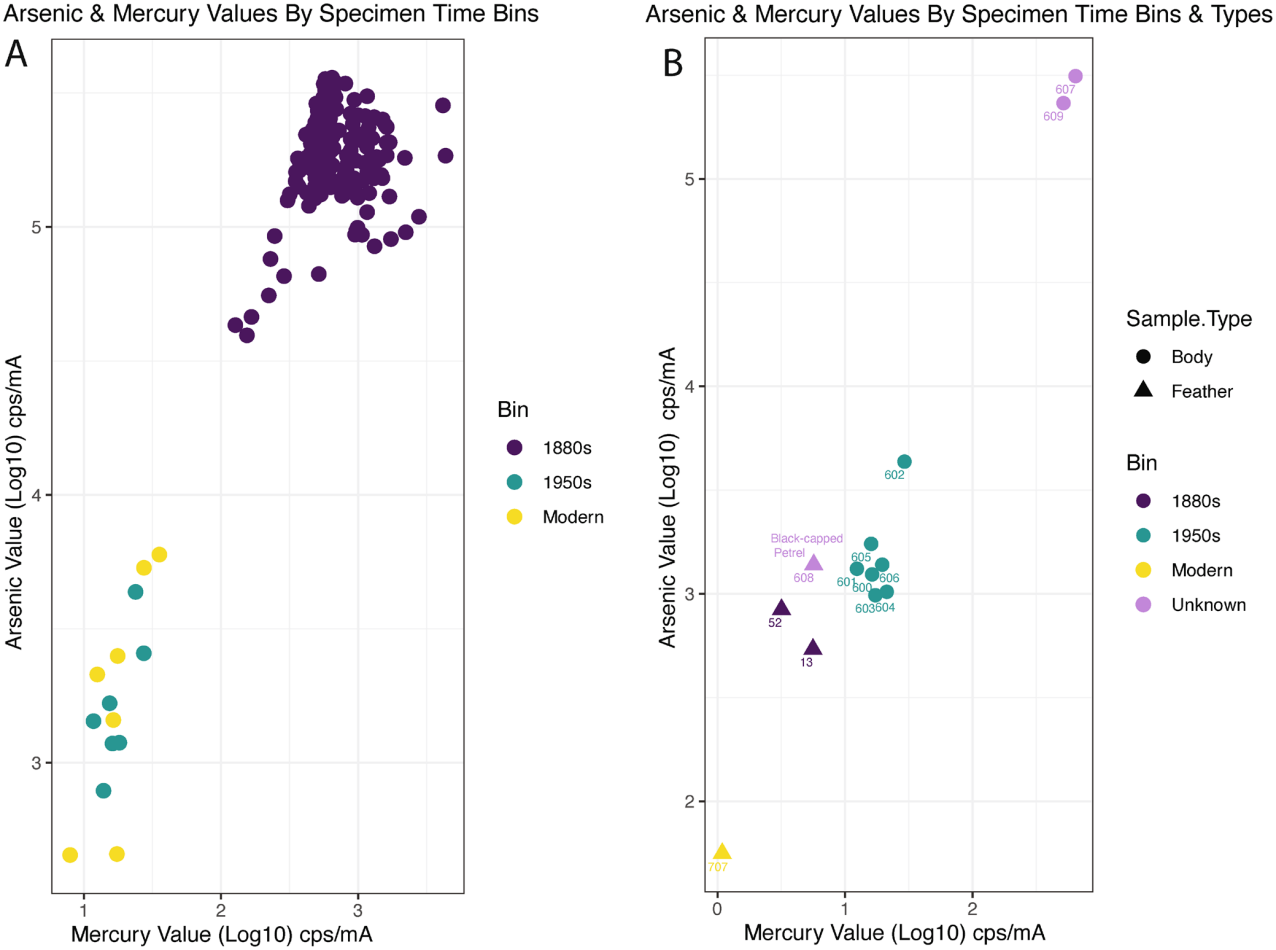
(A) Scatter plot of arsenic and mercury values from whole bird bodies of known specimen age (original labels intact) (*n* 1880s = 198, *n* 1950s = 7, *n* modern [1983–2019] = 7). (B) Relative arsenic and mercury XRF values of individual feathers (triangles; specimens 707, 52, 13, 608) and whole bodies (circles; specimens 600–609). Unknown = specimens 607, 608, 609 of unknown time bin based on label, but suspected 1880s. The Black‐capped Petrel (608) is represented by a single feather. Values reported are in cps/mA, counts per second per milliamp, which is a measurement of peak intensity. Values are presented as log10 due to the magnitude of differences between readings.

### 2.5 Detection of Arsenic and Mercury From Historical Study Skins

Arsenic and mercury were key ingredients in the preservation of bird study skins and taxidermy in the 19th century; soaps and solutions were rubbed into skins to deter pest damage up until the mid‐20th century, and are still detectable in the present day (Farber 1977; Strekopytov et al. 2017; see also “Mr. Balch's Recipe for Poison” from the Fairbanks Museum & Planetarium of Vermont in LeFebvre et al. 2023). As such, there are now human health concerns related to the handling of historic bird specimens (Desjardins 2016; Marcotte et al. 2017). X‐ray fluorescence (XRF) spectrometry has been used to document arsenic and mercury contamination in natural history collections (Marte et al. 2006; Strekopytov et al. 2017). Thus, if the Middlebury *P. hasitata* specimen represents a 19th‐century acquisition, it should have arsenic and mercury levels that match well‐labeled specimens from the Parkhill‐Mead collection, and these would differ significantly from more recent skin preparations that did not include arsenic and mercury (S. Trombulak, personal communication).

As part of ongoing testing for environmental health and safety remediation, we generated a reference set of qualitative arsenic and mercury measurements for over 200 whole birds or individual feathers using the Thermo Scientific ARL Quant'X energy dispersive XRF spectrometer at Middlebury College (see Supporting Information for values). Spectra were collected for 30 s at 28 kV using a thick Pd filter. The machine surface was cleaned with isopropanol after each run to avoid cross‐contamination (see Desjardins 2016; Marcotte et al. 2017; Marte et al. 2006; Strekopytov et al. 2017 for more examples of these methods that can be of use to small museum collections). Values reported are in counts per second per milliamp (cps/mA), which is a measurement of peak intensity. While our XRF results are qualitative and relative rather than the absolute parts per million values of arsenic within each bird, a clear, statistically significant difference emerged between 19th‐century specimens and all others in our collection (Figure 5), with 19th century birds exhibiting arsenic levels several orders of magnitude higher. A Shapiro–Wilk test conducted in R indicated that residuals of our dataset followed a normal distribution. The three groups examined (1880s, 1950s, “modern” 1983–2019) were significantly different (*p* < 0.001, ANOVA). Subsequent Tukey HSD post hoc tests using the R package “multcomp” revealed significant differences between 1880s birds with both 1950s and modern (1983–2019) specimens (*p* < 0.001) and no significant differences between 1950s and modern specimens (*p* > 0.05). These clear differences in arsenic and mercury levels permitted us to use arsenic as a metric for assessing the age of the specimen based on its preparation (Figure 6A). It is also likely that due to past curation practices at Middlebury, even the small number of 1950s and modern specimens have elevated arsenic levels due to cross‐contamination within cabinets, making our results conservative estimates.

As the Black‐capped Petrel specimen (#608) was too large to safely insert into the machine, we opted to sample an individual loose feather from it as well as from three other specimens of waterbirds of known time bin and similar body size (Figure 6B). We compared these feathers to entire body readings of other specimens labeled with values of 600 (i.e., 600–609), most of which were labeled as being from the 1957 Van Vleck collection. A single feather from the Black‐capped Petrel returned values for arsenic and mercury that were on par with values for the entire bodies of 1957 specimens and were consistent with feathers from other 1880s birds (Table 1; Figure 6B). Though the Black‐capped Petrel is labeled as specimen number #608 and there are intervening specimens (#600–606) labeled clearly as originating in Ithaca, New York in 1957, most of the 19th‐century taxidermied mounts fall within the 700 series of the collection. It is clear that the unlabeled specimens 607 and 609 also reflect 19th‐century arsenic practices based on their values (Figure 6B). The numbering system is also discontinuous and reflects the lack of systematic curation attention and the impact of non‐standardized efforts across previous workers in the collection. The value of arsenic for the Black‐capped Petrel, 1382.55 (cps/mA), was higher than either feather from known 1880s birds collected by Parkhill and Mead (Table 1). Our result of heightened inorganic mercury in the Black‐capped Petrel due to 19th‐century curatorial practices (rather than linked to biomagnification and industrial processes in the form of methylmercury) is also consistent with previous studies of pelagic seabird museum specimens (Vo et al. 2011). Therefore, we can reasonably assign specimen #608, the Black‐capped Petrel, to a 19th‐century origin.

**TABLE 1:** Arsenic levels in sampled feathers and whole bodies, as displayed in Figure 6B. Unknown indicates unlabeled tag or missing tag, but suspected 1880s. 1950s specimens are from 1957 based on available tag information.

Specimen #	Species	Common name	Bin	Collector	Arsenic (cps/mA)	Type
13	*Lophodytes cucullatus*	Hooded Merganser	1880s	Mead	540.73	Feather
52	*Fulica americana*	American Coot	1880s	Parkhill	840.03	Feather
600	*Passer domesticus*	House Sparrow	1950s	Van Vleck	1239.84	Body
601	*Molothrus ater*	Brown‐headed Cowbird	1950s	Van Vleck	1320.13	Body
602	*Hirundo rustica*	Barn Swallow	1950s	Van Vleck	4334.59	Body
603	*Passer domesticus*	House Sparrow	1950s	Van Vleck	984.03	Body
604	*Passer domesticus*	House Sparrow	1950s	Van Vleck	1022.2	Body
605	*Quiscalus*	Common Grackle	1950s	Van Vleck	1740.73	Body
606	*Passer domesticus*	House Sparrow	1950s	Van Vleck	1381.41	Body
607	*Haemorhous purpureus*	Purple Finch	Unknown	Unknown	312936.24	Body
608	*Pterodroma hasitata*	Black‐capped Petrel	Unknown	Unknown	1382.55	Feather
609	*Anthus rubescens*	American Pipit	Unknown	Unknown	231,847.98	Body
707	*Bucephala clangula*	Common Goldeneye	“modern” 1983–2019	Walton	56.03	Feather

## 3 Discussion

Geographic distribution and other types of data gleaned from historical museum specimens have facilitated the reconstruction of preindustrial distribution baselines relevant to conservation planning (Grace et al. 2019; Meineke et al. 2019). Vagrancy across a range of avian taxa is markedly increasing as a result global climate change (Jiguet and Barbet‐Massin 2013), and historical data may play a role in contextualizing its conservation significance. In a small population, acute mortality events represented by large‐scale storm wrecking can be of conservation concern (Hass, Hyman, and Semmens 2012, Davis and Watson 2018). The presence of a Black‐capped Petrel from the late 19th century in Vermont is significant because it likely represents the earliest record of the species within New England, as well as one of the earliest inland North American records confirmed by a physical specimen. If concurrent with other specimens from the 1893 hurricane—as is suggested by the independent line of evidence presented by the Vermont Birds Record Committee—it is predated by only two other specimens, one being from an unknown US location in 1821 and another from Quogue, NY, in 1850 (Dutcher 1889; Table 2). This record fills a geographic gap in the known vagrant range of the species (BirdLife International 2018) and contributes to a substantial pool of existing storm wrecking records (48, Hass, Hyman, and Semmens 2012). Such records that predate substantial anthropogenic climate change serve as a basis for interpreting the significance of future storm wrecking incidents; they help better describe the natural range of variation of vagrancy for this species in the absence of anthropogenic climate change, thereby contextualizing the novelty (and potential urgency) of future shifts in vagrancy (Silliman et al. 2018).

**TABLE 2:** Continental North American records of Black‐capped Petrel prior to 1893, as found on the Global Biodiversity Information Facility (GBIF) (GBIF.org 2024).

Date	Location
1823 (no date)	No location, USA
1850, August	Quogue, New York, USA
1893, August 28	Oneida Lake, New York, USA
1893, August 30	Pittsfield, New Hampshire, USA
1893, August 30	Blacksburg, Virginia, USA
1893, September	Cayuga County, New York, USA
1893, October 30	Toronto, Ontario, Canada
1893 (no date)	Halton, Ontario, Canada

The Black‐capped Petrel's present‐day nesting and foraging range is located within the west Atlantic tropical cyclone belt, a zone marked by frequent and powerful hurricanes generated off the west coast of Africa that predominantly occur in late summer and early fall (Hass, Hyman, and Semmens 2012). Satellite tracking has indicated petrels are often able to avoid, precede, or follow tropical cyclones, generally moving to low‐wind areas by either passing around the storm or attempting to reach its calmer eye (Lempidakis et al. 2022; Nourani et al. 2023). However, Black‐capped Petrel storm wrecking is associated with Category 3+ storms that strike the continent directly rather than recurving, prohibiting birds from escaping southwestward along the coast (Hass, Hyman, and Semmens 2012). The International Black‐capped Petrel Conservation Group notes hurricane fallout among the threats nonbreeding adults and immatures of this species face at sea (Wheeler et al. 2021). While the overall level of this threat is rated as low/medium with low uncertainty (Wheeler et al. 2021), scope and severity are rated as medium, and irreversibility of such mortality events is rated as high, with recovery only possible over decades. This area is further noted as an area of data lack, as well as being a threat impacted by climate change (Wheeler et al. 2021).

Current rising sea surface temperatures are anticipated to contribute to stronger and more frequent hurricanes over the coming decades (Hass, Hyman, and Semmens 2012; Vecchi et al. 2021), which may yield novel and intensified patterns of seabird storm wrecking. As the phenomenon of inland fallout is not limited to the Black‐capped Petrel, the intensification of storm wrecking events may be of conservation importance to other Gulf Stream seabirds. Tropicbirds *Phaethon* sp. (Lee and Irvin 1983), other Procellariform taxa (LeGrand Jr 1990), and *Onychoprion* terns (LeGrand Jr 1990; Huang, Bass Jr, and Pimm 2017) are among the taxa associated with inland fallout in the wake of west Atlantic hurricanes. *Pterodroma* storm wrecking has also been recorded away from the Gulf Stream, including in Japan (Mitsuishi, Tominaga, and Hasuo 1964) and Brazil (Bugoni, Sander, and Costa 2007). While limited in scale, these stochastic mortality events must be considered in both historical and modern analyses of population dynamics. The late 19th‐century specimen at Middlebury College is also relevant from a perspective of historic genetic diversity. This individual was collected during a period of broad collapse in Black‐capped Petrel populations (Satgé et al. 2024) and could therefore contain genetic diversity now lost from the species. As ongoing research investigates population structure within the species (Manly et al. 2013), museum specimens may offer new insights into the complex taxonomy and genomic history of the species, potentially with conservation implications for the species and its two forms, as has been the case for a number of recently extinct or extirpated bird species (e.g., Johnson and Dunn 2006).

Museum specimens have long been valued for the insights they can provide into how species respond to environmental and anthropogenic change (Lendemer et al. 2020; Schmitt et al. 2018) and are now regularly considered in conservation planning efforts (e.g., LeFebvre et al. 2023). The COVID‐19 pandemic resulted in increased awareness of the value of museum collections as biorepositories, as natural history specimens have generated a wealth of information about the ecology and evolution of infectious diseases and their vectors (Thompson et al. 2021). While the majority of such research focuses on large collections with thousands of specimens drawn from a global extent, small museum collections, such as the Middlebury College Vertebrate Natural History collection, represent spatially restricted ecological time capsules that can be used to reconstruct baselines of greater relevance to local conservation decision‐making and can facilitate place‐based educational pedagogies (Marsico et al. 2020). The specimens held in small museum collections are often unique due to the specific histories that led to their inclusion in such institutions that were not part of formal government or scientific collecting trips, as is the case for our collection. Based on our unexpected finding, we hope that other Black‐capped Petrel specimens might still surface in small collections held at regional colleges and museums, and we encourage renewed attention to the careful curation of small museum collections to uncover further important surprises and to avoid potential data loss for future researchers.

## Author Contributions

**Oliver W. Patrick:** conceptualization (equal), data curation (equal), formal analysis (equal), funding acquisition (supporting), investigation (lead), methodology (equal), visualization (equal), writing – original draft (lead), writing – review and editing (equal). **Max Chalfin‐Jacobs:** conceptualization (equal), data curation (equal), investigation (equal), writing – review and editing (supporting). **Arthur Lyu:** investigation (equal), methodology (equal). **Jody Smith:** investigation (equal), resources (equal), supervision (equal). **Ellery Foutch:** conceptualization (equal), investigation (equal), methodology (supporting), resources (equal), writing – review and editing (supporting). **Alexis M. Mychajliw:** conceptualization (equal), data curation (equal), funding acquisition (lead), investigation (equal), methodology (equal), project administration (lead), supervision (lead), visualization (equal), writing – original draft (supporting).

## Conflicts of Interest

The authors declare no conflicts of interest.

## Supporting information


Data S1.

## Data Availability

All relevant data are available as tables within the paper itself or can be found within the Supporting Information spreadsheet.
